# Agreement of Electrolyte Measurements Between Arterial Blood Gas Analyzers and Central Laboratories in Critically Ill Patients with Acute Kidney Injury

**DOI:** 10.3390/medsci14030383

**Published:** 2026-07-09

**Authors:** Atthaphong Phongphithakchai, Sitthikorn Thingphom, Aman Tedasen, Chutima Jansakun, Wiyada Kwanhian Klangbud, Jongkonnee Thanasai, Fumitaka Kawakami, Moragot Chatatikun

**Affiliations:** 1 Nephrology Unit, Division of Internal Medicine, Faculty of Medicine, Prince of Songkla University, Songkhla 90110, Thailand; atthaphong.p@psu.ac.th (A.P.); sitthikorn2541@gmail.com (S.T.); 2 School of Allied Health Sciences, Walailak University, Nakhon Si Thammarat 80161, Thailand; aman.te@wu.ac.th (A.T.); chutima.js@wu.ac.th (C.J.); 3 Research Excellence Center for Innovation and Health Products, Walailak University, Nakhon Si Thammarat 80161, Thailand; 4 Medical Technology Program, Faculty of Science, Nakhon Phanom University, Nakhon Phanom 48000, Thailand; wiyadakwanhian@gmail.com; 5 Faculty of Medicine, Mahasarakham University, Mahasarakham 44000, Thailand; jongkonnee@msu.ac.th; 6 Department of Regulatory Biochemistry, Kitasato University Graduate School of Medical Sciences, Sagamihara 252-0373, Japan; kawakami@kitasato-u.ac.jp

**Keywords:** acute kidney injury, arterial blood gas analyzer, point-of-care testing, electrolyte measurement, potassium, critically ill patients

## Abstract

**Background:** Electrolyte disturbances are common in critically ill patients with acute kidney injury (AKI) and often require urgent intervention. Arterial blood gas (ABG) analyzers provide rapid point-of-care electrolyte measurements, but their agreement with central laboratory analyzers in critically ill patients with AKI remains uncertain. This study evaluated the agreement between ABG and central laboratory measurements of sodium (Na^+^), potassium (K^+^), and chloride (Cl^−^). **Methods:** We conducted a retrospective observational study of adult critically ill patients with AKI admitted to a tertiary university hospital ICU between January 2013 and June 2024. Patients with paired electrolyte measurements obtained from an ABG analyzer (ABL800 Basic, Radiometer) and a central laboratory analyzer (Abbott Alinity) within 10 min were included. Agreement was assessed using Bland–Altman analysis and Passing–Bablok regression. **Results:** A total of 1870 critically ill patients with AKI were included. The mean bias (95% limits of agreement [LOA]) between ABG and central laboratory measurements was −0.68 mmol/L (−11.41 to 10.06) for sodium, −0.17 mmol/L (−1.46 to 1.12) for potassium, and 7.18 mmol/L (−4.15 to 18.51) for chloride. Potassium showed the narrowest LOA. Pearson correlation coefficients were 0.736, 0.642, and 0.728 for sodium, potassium, and chloride, respectively. Clinically meaningful discrepancies were observed in 7.0% of sodium, 14.1% of potassium, and 31.4% of chloride measurements. **Conclusions:** ABG-derived sodium and potassium measurements showed relatively good agreement with central laboratory measurements, particularly for potassium. However, clinically meaningful discrepancies occurred in a subset of measurements, suggesting that preanalytical factors and specimen quality may influence measurement reliability. ABG-derived electrolyte measurements may support rapid bedside assessment, whereas central laboratory measurements remain the reference standard.

## 1. Introduction

Acute kidney injury (AKI) is a common complication in critically ill patients and is associated with increased morbidity, mortality, prolonged hospitalization, and healthcare burden [1,2,3,4]. Electrolyte disturbances frequently occur in AKI because of impaired renal excretory function, altered acid-base homeostasis, fluid shifts, and therapeutic interventions, including diuretics and kidney replacement therapy [5,6,7]. Among these abnormalities, disturbances in sodium, potassium, and chloride are particularly clinically relevant because they may rapidly contribute to life-threatening complications such as cardiac arrhythmias, neurologic dysfunction, hemodynamic instability, and worsening organ dysfunction [8,9,10,11,12,13]. Hyperkalemia, in particular, is a medical emergency frequently encountered in critically ill patients with AKI and often requires immediate intervention to prevent fatal cardiac complications [8,12].

Rapid and accurate electrolyte assessment is therefore essential in critically ill patients. Conventional central laboratory testing remains the standard method for electrolyte measurement in most institutions; however, turnaround time may be delayed because of specimen transport and laboratory processing [14,15,16,17,18]. In contrast, arterial blood gas (ABG) analyzers provide rapid point-of-care testing (POCT), allowing electrolyte results to be obtained within minutes and facilitating timely clinical decision-making in emergency and intensive care settings [19,20]. In critically ill patients with AKI, where electrolyte concentrations may change rapidly, immediate laboratory information is crucial for guiding interventions such as potassium-lowering therapy, fluid management, bicarbonate administration, and initiation of kidney replacement therapy.

Despite these advantages, electrolyte measurements obtained from ABG analyzers and central laboratory analyzers may not be directly interchangeable because of methodological differences between the analytical systems. Blood gas analyzers measure electrolytes directly from whole blood using direct ion-selective electrode (ISE) methods, whereas central laboratory analyzers commonly use indirect ISE methods on diluted plasma or serum samples [14,16]. Differences in specimen type, sample preparation, calibration procedures, and analytical techniques may result in systematic bias and clinically meaningful discrepancies [14,19]. Previous investigations have shown that discrepancies between point-of-care and central laboratory electrolyte measurements may influence clinical interpretation, including acid–base assessment and electrolyte management [16].

Previous studies evaluating agreement between blood gas and central laboratory electrolyte measurements have yielded conflicting results, with some demonstrating acceptable agreement and others reporting clinically significant discrepancies; thus, no definitive conclusion regarding their interchangeability has been established [14,21,22]. Given the limited evidence in critically ill patients with AKI, this study aimed to evaluate the agreement between arterial blood gas analyzer and central laboratory measurements of sodium, potassium, and chloride concentrations in this high-risk population.

## 2. Materials and Methods

### 2.1. Study Design and Population

This retrospective observational study was conducted in the intensive care unit (ICU) of Songklanagarind Hospital, a tertiary care university hospital in southern Thailand. Adult critically ill patients admitted to the ICU between 1 January 2013 and 30 June 2024, were screened for eligibility. 

Ethical approval was granted by the Human Research Ethics Committee, Faculty of Medicine, Prince of Songkla University (REC.67-526-14-1) on 26 November 2024. The requirement for informed consent was waived due to the retrospective nature of the study.

Patients were included if they met all of the following criteria: (1) age ≥ 18 years; (2) ICU admission; (3) diagnosis of AKI according to the Kidney Disease: Improving Global Outcomes (KDIGO) 2012 criteria; and (4) availability of paired electrolyte measurements, including sodium (Na^+^), potassium (K^+^), and chloride (Cl^−^), obtained using both an ABG analyzer and a central laboratory analyzer, with an interval between measurements not exceeding 10 min. Patients were excluded if they (1) had received chronic dialysis before ICU admission; (2) were pregnant; (3) had blood gas electrolyte measurements obtained from venous rather than arterial blood samples; (4) had central laboratory electrolyte measurements performed on hemolyzed specimens; or (5) received any intervention potentially affecting electrolyte concentrations during the 10-min interval between paired measurements, including electrolyte replacement, bicarbonate administration, potassium-lowering therapy, fluid administration for electrolyte correction, or kidney replacement therapy. To minimize repeated-measurement bias and ensure independence of observations, only the first eligible paired electrolyte measurement from each patient was included in the analysis.

### 2.2. Definitions and Outcomes

Acute kidney injury was defined according to the KDIGO 2012 criteria as an increase in serum creatinine of ≥0.3 mg/dL within 48 h, an increase in serum creatinine to ≥1.5 times baseline within the prior 7 days, or a urine output < 0.5 mL/kg/h for at least 6 h. AKI severity was classified into stages 1–3 according to KDIGO criteria at the time of blood collection. The primary outcome was the agreement between electrolyte measurements obtained from the arterial blood gas analyzer and the central laboratory analyzer for sodium, potassium, and chloride concentrations.

### 2.3. Data Collection

Clinical and laboratory data were retrospectively extracted from the electronic medical record and laboratory information systems. Arterial blood gas samples were obtained from arterial blood specimens, either through arterial catheter sampling or direct arterial puncture according to routine ICU practice and were analyzed using balanced heparinized blood gas syringes. Arterial blood gas samples were analyzed using the ABL800 Basic blood gas analyzer (Radiometer, Copenhagen, Denmark), which measures electrolytes using a direct ISE method on whole blood samples. Central laboratory electrolyte measurements were performed using the Abbott Alinity analyzer (Abbott Laboratories, Abbott Park, IL, USA) utilizing an indirect ISE method on heparinized plasma specimens collected according to routine hospital laboratory protocols. Only paired electrolyte measurements obtained within 10 min were included to minimize temporal physiological variation between measurements. In routine clinical practice, central laboratory turnaround time is generally longer than ABG testing because of specimen transport and laboratory processing; however, exact turnaround time data were not consistently available retrospectively and therefore were not formally analyzed. No intervention intended to correct electrolyte abnormalities was allowed during this interval to minimize physiological variation between measurements.

The following variables were collected: age, sex, body weight, baseline serum creatinine, maximum serum creatinine during hospitalization, AKI stage at the time of blood collection, presence of shock, sepsis status (sepsis vs. non-sepsis), invasive mechanical ventilation, central venous catheter placement, vasopressor use, extracorporeal membrane oxygenation (ECMO) support, and renal replacement therapy (RRT).

### 2.4. Statistical Analysis

Continuous variables were expressed as mean ± standard deviation (SD) for normally distributed data or median with interquartile range (IQR) for non-normally distributed data, as appropriate. Categorical variables were summarized as frequency and percentage. Agreement between electrolyte measurements obtained from the arterial blood gas (ABG) analyzer and central laboratory analyzer was assessed separately for Na^+^, K^+^, and Cl^−^ concentrations using Bland–Altman analysis and Passing–Bablok regression analysis, which are established statistical methods for method-comparison studies. Bland–Altman analysis was performed to evaluate agreement between the two measurement methods by calculating the mean bias and 95% limits of agreement (LOA), defined as the mean difference ±1.96 standard deviations of the differences. Bland–Altman plots were constructed to visually assess the distribution of measurement differences and identify potential trends in disagreement across the range of electrolyte concentrations. Passing–Bablok regression analysis, a non-parametric regression method robust to outliers and measurement error, was used to evaluate potential systematic and proportional differences between the ABG analyzer and central laboratory measurements. Regression equations and 95% confidence intervals (CI) for the intercept and slope were calculated. Pearson correlation coefficients were additionally reported to describe the strength of linear association between measurement methods. In addition, an exploratory discrepancy analysis was performed to identify potentially clinically significant differences between methods. Predefined clinically significant discrepancies were defined as absolute differences of >10 mmol/L for sodium, >1.0 mmol/L for potassium, and >10 mmol/L for chloride. The number and percentage of paired measurements exceeding these thresholds were calculated. A two-sided *p*-value < 0.05 was considered statistically significant. All statistical analyses were performed using R software version 4.4.0 (R Foundation for Statistical Computing, Vienna, Austria).

## 3. Results

### 3.1. Patient Characteristics

**Figure 1:**
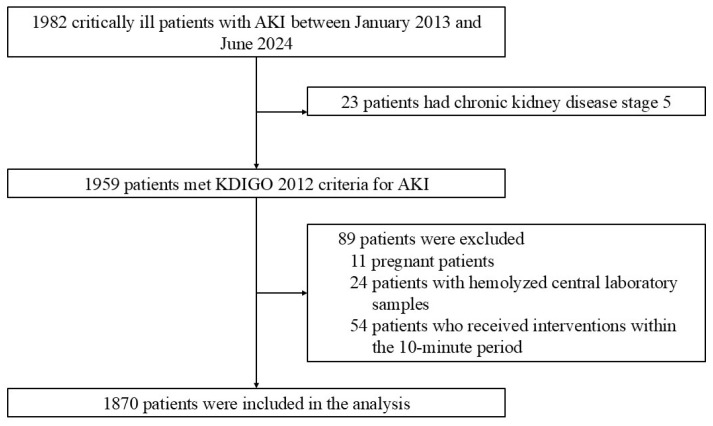
Study population flow diagram.

A total of 1982 critically ill patients with AKI admitted between January 2013 and June 2024 were initially screened for eligibility. After excluding 23 patients with chronic kidney disease stage 5, 1959 patients met the KDIGO 2012 criteria for AKI. Subsequently, 89 patients were excluded, including 11 pregnant patients, 24 patients with hemolyzed central laboratory samples, and 54 patients who received electrolyte-related interventions within the 10-min interval between paired measurements. Finally, 1870 patients were included in the analysis (Figure 1).

Baseline characteristics of the study population are summarized in Table 1. The mean age was 63.7 ± 16.4 years, and 1189 patients (63.6%) were male. The mean body weight was 61.3 ± 13.4 kg, and 761 patients (40.7%) had a history of smoking. Regarding ICU admission, most patients were admitted to the medical intensive care unit (MICU) (43.3%) or surgical intensive care unit (SICU) (40.8%), followed by the coronary care unit (CCU) (8.7%) and cardiovascular intensive care unit (ICU-CVT) (7.2%). Common comorbidities included hypertension (60.5%), diabetes mellitus (54.9%), dyslipidemia (52.5%), and chronic kidney disease (52.0%). Cardiovascular and cerebrovascular diseases were present in 43.5% and 30.8% of patients, respectively, while 7.8% had chronic liver disease. The median baseline serum creatinine was 1.9 mg/dL (IQR 1.8), whereas the mean serum creatinine at AKI diagnosis was 3.7 ± 4.2 mg/dL. At the time of blood collection, AKI stage 3 was the most common stage (48.9%), followed by stage 1 (40.8%) and stage 2 (10.3%). Sepsis was present in 350 patients (18.7%). Most patients required intensive supportive therapies, including mechanical ventilation (85.8%), central venous catheter insertion (83.4%), and vasopressor support (51.8%). In addition, 62 patients (3.3%) required ECMO and 583 patients (31.2%) underwent RRT.

### 3.2. Agreement Between Arterial Blood Gas and Central Laboratory Electrolyte Measurements

**Figure 2:**
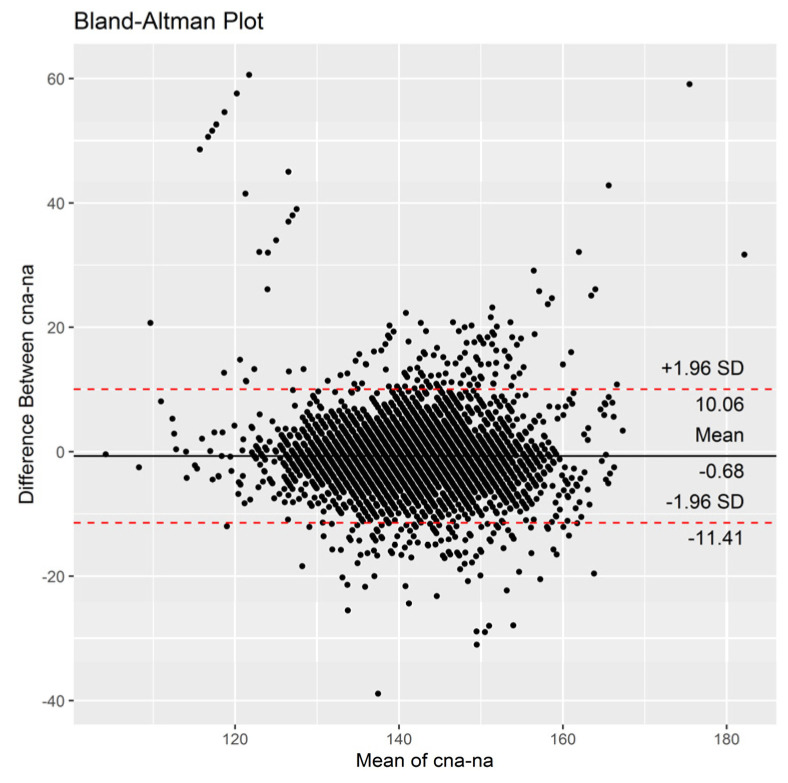
Bland–Altman plots comparing electrolyte measurements obtained from the arterial blood gas (ABG) analyzer and central laboratory analyzer for sodium concentrations. The solid horizontal line represents the mean bias between measurement methods, while the dashed lines indicate the 95% limits of agreement (LOA), calculated as the mean difference ±1.96 standard deviations. Positive values indicate higher electrolyte concentrations measured by the ABG analyzer relative to the central laboratory analyzer.

**Figure 3:**
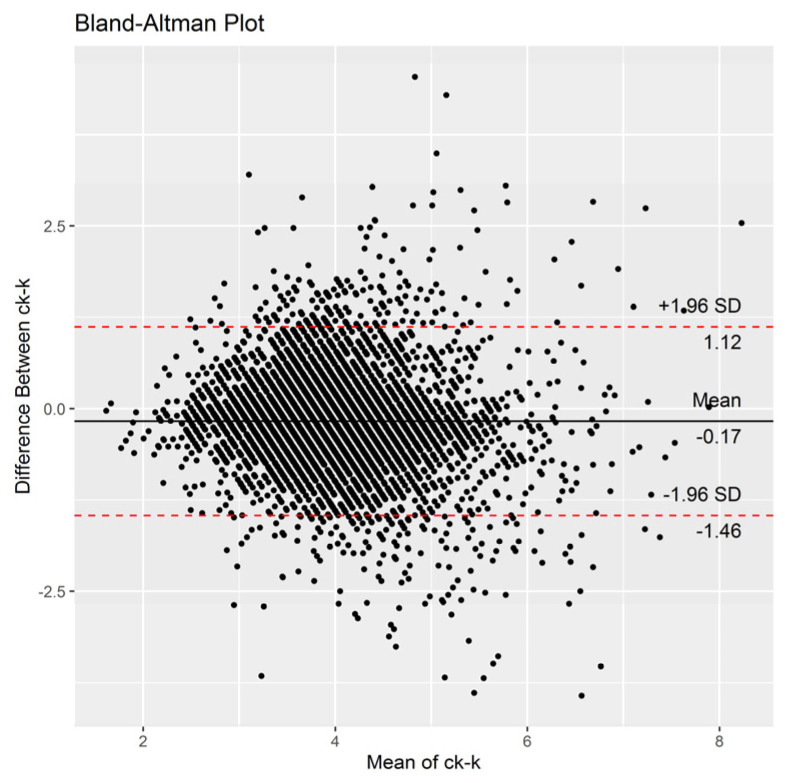
Bland–Altman plots comparing electrolyte measurements obtained from the arterial blood gas (ABG) analyzer and central laboratory analyzer for potassium concentrations. The solid horizontal line represents the mean bias between measurement methods, while the dashed lines indicate the 95% limits of agreement (LOA), calculated as the mean difference ±1.96 standard deviations. Positive values indicate higher electrolyte concentrations measured by the ABG analyzer relative to the central laboratory analyzer.

Agreement between electrolyte measurements obtained from the ABG analyzer and central laboratory analyzer for Na^+^, K^+^, and Cl^−^ concentrations was evaluated using Bland–Altman analysis and Passing–Bablok regression analysis (Figure 2 and Figure 3).

#### 3.2.1. Bland–Altman Analysis

**Figure 4:**
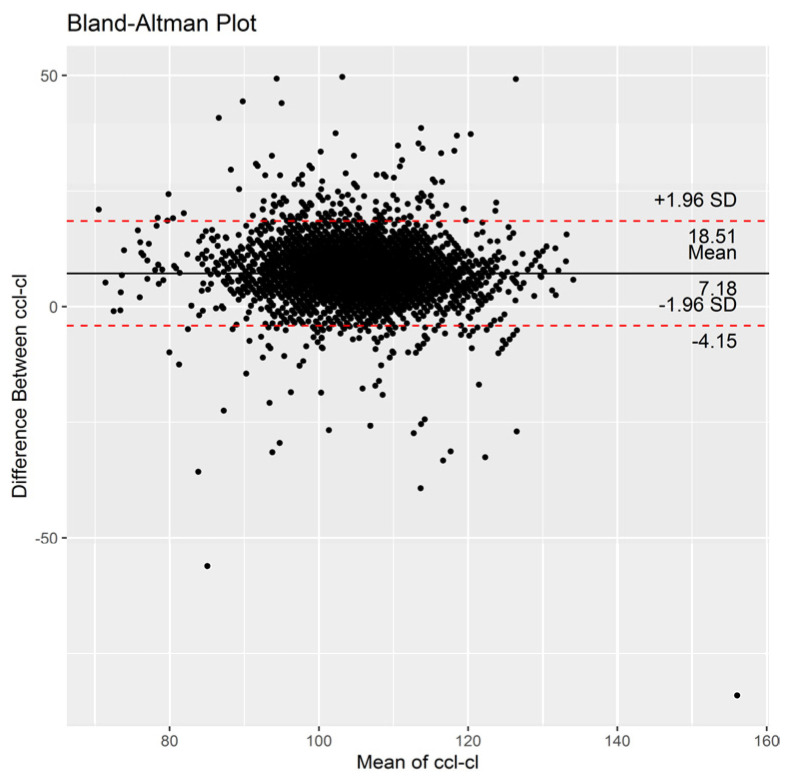
Bland–Altman plots comparing electrolyte measurements obtained from the arterial blood gas (ABG) analyzer and central laboratory analyzer for chloride concentrations. The solid horizontal line represents the mean bias between measurement methods, while the dashed lines indicate the 95% limits of agreement (LOA), calculated as the mean difference ±1.96 standard deviations. Positive values indicate higher electrolyte concentrations measured by the ABG analyzer relative to the central laboratory analyzer.

Bland–Altman analysis demonstrated varying degrees of agreement between electrolyte measurements obtained from the ABG analyzer and central laboratory analyzer (Figure 2). The mean bias and 95% LOA for sodium, potassium, and chloride measurements are shown in Figure 2, Figure 3 and Figure 4. For sodium concentrations, the mean bias between methods was −0.68 mmol/L, indicating slightly lower sodium values obtained from the ABG analyzer compared with the central laboratory analyzer. The 95% LOA ranged from −11.41 to 10.06 mmol/L (Figure 2). Most data points were distributed closely around the mean bias line, although several observations extended beyond the limits of agreement, particularly at extreme sodium concentrations. No obvious trend of increasing disagreement across the measurement range was observed. For potassium concentrations, the mean bias was −0.17 mmol/L, suggesting only a small difference between measurements obtained from the two analytical methods. The 95% LOA ranged from −1.46 to 1.12 mmol/L (Figure 3), representing the narrowest agreement interval among the evaluated electrolytes. Data points were symmetrically distributed around the mean bias, with relatively limited dispersion across the concentration range. For chloride concentrations, the mean bias was 7.18 mmol/L, indicating higher chloride values measured by the ABG analyzer than by the central laboratory analyzer. The 95% LOA ranged from −4.15 to 18.51 mmol/L (Figure 4), demonstrating wider variability compared with sodium and potassium measurements. Greater dispersion of differences was observed throughout the chloride measurement range, with a broader spread of observations around the mean bias. Overall, sodium and potassium measurements demonstrated smaller mean differences and narrower variability between analytical methods, whereas chloride measurements exhibited greater mean bias and wider limits of agreement. To further evaluate clinically relevant disagreement, an additional discrepancy analysis was performed using predefined clinically significant thresholds. Substantial discrepancies were observed in 131/1870 (7.0%) sodium measurements with an absolute difference > 10 mmol/L, 264/1870 (14.1%) potassium measurements with an absolute difference > 1.0 mmol/L, and 588/1870 (31.4%) chloride measurements with an absolute difference > 10 mmol/L.

#### 3.2.2. Passing–Bablok Regression Analysis

**Figure 5:**
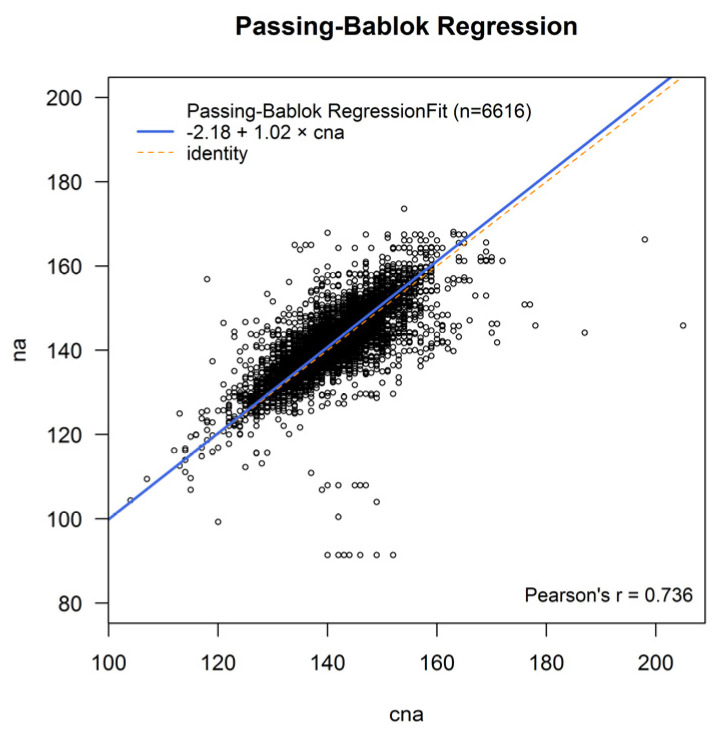
Passing–Bablok regression analysis comparing electrolyte measurements obtained from the arterial blood gas (ABG) analyzer and central laboratory analyzer for sodium. Solid lines represent Passing–Bablok regression lines and dashed lines indicate the identity line. Abbreviations: cna, central laboratory sodium concentration.

**Figure 6:**
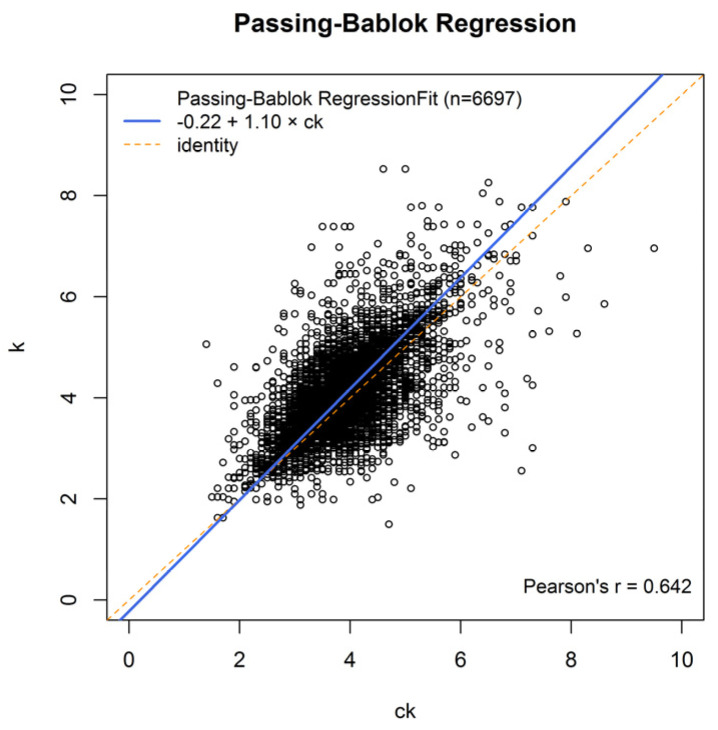
Passing–Bablok regression analysis comparing electrolyte measurements obtained from the arterial blood gas (ABG) analyzer and central laboratory analyzer for potassium. Solid lines represent Passing–Bablok regression lines and dashed lines indicate the identity line. Abbreviations: cck, central laboratory potassium concentration.

**Figure 7:**
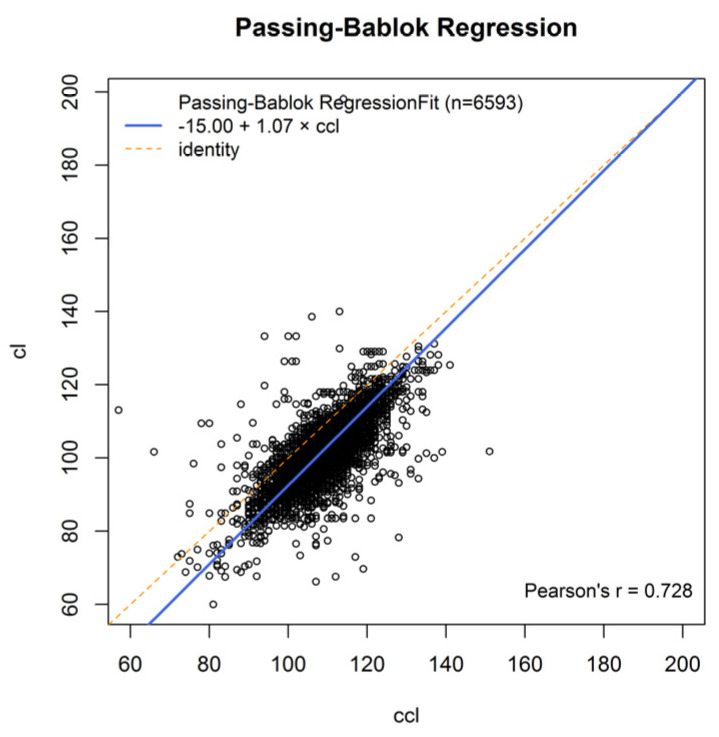
Passing–Bablok regression analysis comparing electrolyte measurements obtained from the arterial blood gas (ABG) analyzer and central laboratory analyzer for chloride concentrations. Solid lines represent Passing–Bablok regression lines and dashed lines indicate the identity line. Abbreviations: ccl, central laboratory chloride concentration.

Passing–Bablok regression analysis was performed to evaluate systematic and proportional differences between electrolyte measurements obtained from the ABG analyzer and central laboratory analyzer. For sodium, the Passing–Bablok regression equation was y = −2.18 + 1.02x (Figure 5). The regression line closely approximated the line of identity throughout the measurement range. The Pearson correlation coefficient between ABG and central laboratory sodium measurements was 0.736, indicating a moderate-to-strong positive linear association. For potassium, the regression equation was y = −0.22 + 1.10x (Figure 6). Compared with sodium, a slightly greater deviation from the identity line was observed at higher potassium concentrations. The Pearson correlation coefficient for potassium measurements was 0.642. For chloride, the regression equation was y = −15.00 + 1.07x (Figure 7). Greater deviation from the identity line was observed across the measurement range compared with sodium and potassium measurements. The Pearson correlation coefficient for chloride measurements was 0.728.

Across all electrolytes, positive linear associations were observed between measurements obtained from the ABG analyzer and central laboratory analyzer, although the degree of agreement and variability differed among sodium, potassium, and chloride concentrations.

## 4. Discussion

In this retrospective study of critically ill patients with AKI, sodium and potassium measurements obtained from the ABG analyzer demonstrated relatively small mean differences compared with central laboratory measurements, whereas chloride concentrations showed greater systematic variation. Potassium exhibited the narrowest limits of agreement among the evaluated electrolytes, suggesting closer agreement between analytical methods. Given the shorter turnaround time of ABG testing compared with central laboratory analysis and the high prevalence of severe hyperkalemia in critically ill AKI patients, ABG-derived sodium and particularly potassium measurements may provide clinically useful information for rapid bedside decision-making. However, the wider variability observed for sodium and the greater bias in chloride measurements suggest that caution is warranted when interpreting ABG-derived electrolyte values as interchangeable with central laboratory measurements.

Our findings are generally consistent with previous studies evaluating agreement between blood gas analyzers and central laboratory systems, although reported results remain heterogeneous [14,15,16,20,23,24]. Several studies have reported acceptable agreement between ABG-derived sodium and potassium measurements and central laboratory values. In a two-center retrospective analysis, Xie et al. reported high agreement between blood gas analyzers and dry chemistry analyzers for sodium and potassium, particularly when plasma was used as the reference specimen [23]. Similarly, Marija et al. found that sodium and potassium measurements met predefined interchangeability criteria, supporting their use in emergency and critical care settings [20]. These findings are consistent with our observation of relatively small mean differences for sodium and potassium, particularly potassium, which demonstrated the narrowest limits of agreement.

In contrast, other studies have reported clinically relevant disagreement between measurement systems, particularly in critically ill populations. Morimatsu et al. demonstrated significant differences in sodium and chloride concentrations between point-of-care and central laboratory measurements in ICU patients, resulting in altered anion gap and strong ion difference calculations [16]. Notably, greater disagreement was observed for sodium and chloride than potassium, which is consistent with our findings, particularly regarding chloride measurements. The greater variability observed in chloride measurements in our study may be explained by methodological differences between analyzers. The ABL800 Basic blood gas analyzer uses a direct ISE technique on whole blood, whereas the Abbott Alinity central laboratory analyzer uses an indirect ISE method after sample dilution. Such analytical differences may contribute to systematic variation in electrolyte measurements, particularly in critically ill patients with altered plasma composition [16,23]. In particular, abnormal plasma protein or lipid concentrations may influence indirect ISE measurements because electrolyte concentrations are estimated after sample dilution, whereas direct ISE methods measure ion activity in undiluted whole blood. Therefore, conditions such as hypoalbuminemia, dysproteinemia, or hyperlipidemia may contribute to discrepancies between analytical methods in critically ill patients. Notably, direct ISE measurements obtained from blood gas analyzers may more closely reflect physiologically active ion concentrations in altered plasma states [19]. In addition, heparinized blood gas sampling and dynamic acid–base disturbances in critically ill AKI patients may further contribute to chloride variability [16].

Our findings differ somewhat from pediatric and emergency department studies reporting insufficient agreement between blood gas and laboratory measurements. For example, Konuksever et al. reported that sodium and potassium measurements could not be used interchangeably in pediatric emergency patients because agreement limits exceeded clinically acceptable thresholds [24]. An important finding of our study was the presence of a substantial number of electrolyte measurements with large discrepancies between ABG and central laboratory methods, despite relatively small mean bias for sodium and potassium. Such discordant values are unlikely to be explained solely by analytical imprecision because both analytical platforms generally demonstrate low coefficients of variation for electrolyte measurements. Instead, these discrepancies may reflect real-world preanalytical variability frequently encountered in critically ill settings. Potential contributors include contamination from intravenous fluids, specimen collection through vascular access devices, line flushing effects, delayed specimen handling, or altered plasma composition. In critically ill patients with AKI, where multiple infusions, vasopressors, and invasive vascular access are common, obtaining an uncontaminated specimen may substantially influence measurement validity. Therefore, clinically important disagreement between methods may depend not only on analytical technique but also on specimen quality and collection conditions. Indeed, specimen quality and preanalytical factors may have a greater influence on measurement reliability in routine clinical practice than analytical performance differences between platforms. In addition, differences in ICU populations may potentially influence the degree of agreement between ABG and central laboratory electrolyte measurements. Patients admitted to different ICU settings, including the MICU, CCU, SICU, and ICU-CVT, may vary substantially in illness severity, hemodynamic instability, vasopressor exposure, acid–base disturbances, extracorporeal support, and fluid administration.

The findings of this study have important implications for the management of critically ill patients with AKI, in whom electrolyte abnormalities frequently require urgent intervention. Given the faster turnaround time of ABG testing compared with central laboratory analysis, ABG-derived potassium measurements may be particularly useful for rapid bedside assessment, especially in patients with suspected severe hyperkalemia where delayed treatment may have serious consequences. Sodium measurements also demonstrated relatively small mean differences and may assist early clinical assessment while awaiting confirmatory laboratory results. Nevertheless, caution remains warranted when interpreting ABG-derived electrolyte values as directly interchangeable with central laboratory measurements. Although sodium and potassium demonstrated relatively small mean biases, clinically important outliers remained present, particularly for sodium and chloride measurements. An additional discrepancy analysis using predefined clinically significant thresholds identified substantial discrepancies in approximately 7.0% of sodium measurements, 14.1% of potassium measurements, and 31.4% of chloride measurements. These findings indicate that, despite acceptable overall agreement, clinically meaningful disagreement may occur in a subset of patients and could potentially influence clinical interpretation and management decisions. These discrepancies may reflect preanalytical factors and specimen validity rather than analytical method alone. Therefore, unexpected electrolyte values particularly those discordant with the clinical picture should prompt consideration of specimen-related issues and repeat testing when clinically indicated.

This study has several strengths. First, we evaluated agreement between ABG and central laboratory electrolyte measurements in a large cohort of critically ill patients with AKI, a population frequently affected by severe electrolyte disturbances yet underrepresented in previous studies. Second, paired measurements were restricted to a ≤10-min interval, and patients receiving electrolyte-correcting interventions during this period were excluded to minimize physiological variation between tests. Third, only one paired sample per patient was included to reduce repeated-measurement bias and ensure independence of observations. Finally, agreement was assessed using both Bland–Altman analysis and Passing–Bablok regression, which are established statistical methods for method-comparison studies.

Several limitations should be acknowledged. First, this was a single-center retrospective study, which may limit generalizability to institutions using different analytical platforms or laboratory workflows. Second, differences in analyzer technology, calibration methods, and anticoagulant composition may influence agreement and limit applicability to other settings. Because simultaneous serum protein and lipid measurements were not consistently available at the exact time of paired electrolyte sampling, we were unable to evaluate the influence of abnormal protein or lipid concentrations on discrepancies between direct and indirect ISE measurements. Third, although efforts were made to minimize temporal variation, physiological changes may still have occurred between paired sample collections in critically ill patients. In addition, detailed information regarding specimen collection, including the exact sampling source (arterial catheter versus direct arterial puncture), discard volumes, and flushing procedures, was not consistently available because of the retrospective study design. Therefore, we could not determine the relative contribution of preanalytical factors to the observed discrepancies between POCT and central laboratory measurements. Finally, subgroup analyses according to electrolyte severity or acid–base status were not performed and warrant further investigation.

## 5. Conclusions

In conclusion, electrolyte measurements obtained from arterial blood gas analyzers demonstrated variable agreement with central laboratory measurements in critically ill patients with AKI. Sodium and potassium showed relatively small mean differences, with potassium demonstrating the closest agreement, supporting its potential role in rapid bedside assessment. However, variability between methods remained, particularly for sodium, while chloride demonstrated greater systematic bias and should be interpreted cautiously. Although ABG-derived electrolyte measurements may facilitate early clinical decision-making, central laboratory measurements remain the reference standard for definitive evaluation. Importantly, clinically meaningful discrepancies may reflect specimen quality and preanalytical factors rather than analytical method alone, emphasizing the importance of obtaining valid specimens when interpreting electrolyte measurements in critically ill patients.

## Figures and Tables

**Table 1:** Baseline characteristics of patients.

Characteristic	N = 1870
Male sex	1189 (63.6)
Age, years	63.7 ± 16.4
Weight, kg	61.3 ± 13.4
Smoking	761 (40.7)
ICU admission	
- MICU - CCU - SICU - ICU-CVT	810 (43.3) 163 (8.7) 763 (40.8) 134 (7.2)
Co-morbidity	
- Hypertension - Diabetic mellitus - Dyslipidemia - Chronic kidney disease - Coronary heart disease - Cerebrovascular disease - Chronic liver disease	1132 (60.5) 1028 (54.9) 983 (52.5) 974 (52.0) 814 (43.5) 576 (30.8) 147 (7.8)
Baseline Cr, mg/dL	1.9 (1.8)
Cr at diagnosis of AKI, mg/dL	3.7 (4.2)
Stage of AKI	
- Stage 1 - Stage 2 - Stage 3	764 (40.8) 193 (10.3) 914 (48.9)
Sepsis	350 (18.7)
Central venous catheter insertion	1560 (83.4)
Mechanical ventilatory support	1604 (85.8)
Vasopressor use	969 (51.8)
ECMO use	62 (3.3)
RRT use during ICU stay *	583 (31.2)

Continuous data are demonstrated as mean ± SD; categorical data are demonstrated as count (percentage). AKI: acute kidney injury; Cr: creatinine; ECMO: extracorporeal membrane oxygenaton; CCU: coronary care unit; MICU: medical intensive care unit; ICU: intensive care unit; ICU-CVT: cardiovascular intensive care unit; RRT: renal replacement therapy; SICU: surgical intensive care unit. * RRT use refers to acute renal replacement therapy initiated during ICU admission after AKI diagnosis and does not include maintenance dialysis prior to ICU admission.

## Data Availability

The data presented in this study are available within the article. Further inquiries can be directed to the corresponding author.

## Abbreviations

The following abbreviations are used in this manuscript:

ABG	Arterial blood gas
AKI	Acute kidney injury
CCU	Coronary care unit
Cl^-^	Chloride
Cr	Creatinine
ECMO	Extracorporeal membrane oxygenation
ICU	Intensive care unit
ICU-CVT	Cardiovascular intensive care unit
ISE	Ion-selective electrode
KDIGO	Kidney Disease: Improving Global Outcomes
K^+^	Potassium
MICU	Medical intensive care unit
Na^+^	Sodium
POCT	Point-of-care testing
RRT	Renal replacement therapy
SICU	Surgical intensive care unit
